# Potential clinical implications of CD4^+^CD26^high^ T cells for nivolumab treated melanoma patients

**DOI:** 10.1186/s12967-023-04184-6

**Published:** 2023-05-11

**Authors:** Domenico Galati, Serena Zanotta, Mariaelena Capone, Gabriele Madonna, Domenico Mallardo, Marilena Romanelli, Ester Simeone, Lucia Festino, Francesca Sparano, Rosa Azzaro, Rosaria De Filippi, Antonio Pinto, Chrystal M. Paulos, Paolo A. Ascierto

**Affiliations:** 1 Istituto Nazionale Tumori IRCCS Fondazione “G. Pascale”, Naples, Italy; 2 Dipartimento di Medicina Clinica e Chirurgia, Università Degli Studi Federico II, Naples, Italy; 3 Division of Surgical Oncology, Department of Surgery, Emory University, Atlanta, GA USA; 4 Department of Microbiology and Immunology, Winship Cancer Institute, Emory University, Atlanta, GA USA; 5 Hematology-Oncology and Stem Cell Transplantation Unit, Istituto Nazionale Tumori IRCCS Fondazione “G. Pascale”, Naples, Italy; 6 Melanoma Cancer Immunotherapy and Innovative Therapy Unit, Istituto Nazionale Tumori IRCCS Fondazione “G. Pascale”, Naples, Italy

**Keywords:** Melanoma, Nivolumab, PD1-Ab, Immunotherapy, CD26, Flow cytometry, Cancer biomarkers

## Abstract

**Background:**

Nivolumab is an anti-PD1 antibody that has dramatically improved metastatic melanoma patients’ outcomes. Nevertheless, many patients are resistant to PD-1 inhibition, occasionally experiencing severe off-target immune toxicity. In addition, no robust and reproducible biomarkers have yet been validated to identify the correct selection of patients who will benefit from anti-PD-1 treatment avoiding unwanted side effects. However, the strength of CD26 expression on CD4^+^ T lymphocytes permits the characterization of three subtypes with variable degrees of responsiveness to tumors, suggesting that the presence of CD26-expressing T cells in patients might be a marker of responsiveness to PD-1-based therapies.

**Methods:**

The frequency distribution of peripheral blood CD26-expressing cells was investigated employing multi-parametric flow cytometry in 69 metastatic melanoma patients along with clinical characteristics and blood count parameters at baseline (W0) and compared to 20 age- and sex-matched healthy controls. Percentages of baseline CD4^+^CD26^high^ T cells were correlated with the outcome after nivolumab treatment. In addition, the frequency of CD4^+^CD26^high^ T cells at W0 was compared with those obtained after 12 weeks (W1) of therapy in a sub-cohort of 33 patients.

**Results:**

Circulating CD4^+^CD26^high^ T cells were significantly reduced in melanoma patients compared to healthy subjects (p = 0.001). In addition, a significant association was observed between a low baseline percentage of CD4^+^CD26^high^ T cells (< 7.3%) and clinical outcomes, measured as overall survival (p = 0.010) and progression-free survival (p = 0.014). Moreover, patients with clinical benefit from nivolumab therapy had significantly higher frequencies of circulating CD4^+^CD26^high^ T cells than patients with non-clinical benefit (p = 0.004) at 12 months. Also, a higher pre-treatment proportion of circulating CD4^+^CD26^high^ T cells was correlated with Disease Control Rate (p = 0.014) and best Overall Response Rate (p = 0.009) at 12 months. Interestingly, after 12 weeks (W1) of nivolumab treatment, percentages of CD4^+^CD26^high^ T cells were significantly higher in comparison with the frequencies measured at W0 (p < 0.0001), aligning the cell counts with the ranges seen in the blood of healthy subjects.

**Conclusions:**

Our study firstly demonstrates that peripheral blood circulating CD4^+^CD26^high^ T lymphocytes represent potential biomarkers whose perturbations are associated with reduced survival and worse clinical outcomes in melanoma patients.

## Background

The incidence of malignant melanoma in Europe ranges from 3 to 5 per 100,000 per year in Mediterranean countries and from 12 to 25 per 100,000 per year in Nordic countries, with a predicted rising trend of diagnosis in the future [1]. The therapeutic approach and prognosis of patients with advanced-stage or metastatic melanoma have changed over the past decade. In particular new molecules such as immune checkpoint inhibitors (ICIs) targeting the Cytotoxic T-Lymphocyte Antigen 4 (CTLA-4) and Programmed Death 1 (PD-1)/Ligand-1 (PD-L1) pathways have dramatically improved the outcome for patients with metastatic melanoma, with median overall survival (OS) now being extended to more than five years [2–8]. Nevertheless, many patients are resistant to PD-1 inhibition upfront, occasionally experiencing severe off-target immune toxicity [9]. Detection of robust, ‘easy to assay’ and reproducible biomarkers could support optimal strategies to enhance the efficacy of ICIs while avoiding unwanted side effects, leading to identifying the correct selection of patients who will benefit from anti-PD-1 monoclonal antibody (mAb) treatment. In recent years, some reports have shown that low lactate dehydrogenase (LDH), tumor burden, neutrophil to lymphocyte ratio, an absent or limited brain disease, and high relative eosinophil to lymphocyte counts could collectively correlate with better OS in melanoma patients treated with anti-PD-1 agents [10, 11]. Hitherto, none of the candidate biomarkers identified have shown predictive capacities to define suitable patients better and to assist them in providing treatment according to the best trade-off of toxicity to efficacy [12].

Over the years, cumulative evidence has revealed that T cell subsets optimally elicit durable memory responses to tumors. Although CD8^+^ T lymphocytes have shown anti-cancer clinical promise [13], limited data have been reported on the role of human CD4^+^ T cell subsets in tumor immunity. Interestingly, a CD4^+^ T cell population with remarkable stemness properties that naturally migrate to tumors was discovered in the context of adoptive immunotherapy, but their impact on ICI therapy remains unknown. In the context of Chimeric Antigen Receptors (CAR) and Tumor-infiltrating lymphocytes (TIL) therapy, a study unveiled that the different intensities of CD26 expression identified three distinct human T CD4^+^ helper cells: regulatory, naive, and stem memory, with varying levels of anti-tumor activity [14]. Follow-up work further revealed that T cells expressing the highest level of CD26—termed CD26^high^CD4^+^ T cell, could regress large established murine and human tumors to a greater extent than conventional helper T cells, including T helper (T h)1, Th2, Th17 and even bulk CD4^+^ T cells [15]. This data might suggest that CD26-expressing T cells in patients might be a marker of responsiveness to various types of immunotherapy, including PD-1 based therapies.

The molecule CD26 is a 110 kDa type II glycoprotein anchored to the membrane, with dipeptidyl peptidase 4 (DPP4) enzymatic activity, expressed by various cells involved in immunological processes and tumor regulation. In particular, CD26 enhances T cell activation, playing a pivotal role in lymphocyte function [16, 17]. Moreover, CD26 can promote T-cell proliferation through its interaction with adenosine deaminase (ADA), a key enzyme catalyzing the irreversible deamination of extracellular immunosuppressive adenosine into inosine, a carbon source that was recently reported to reduce tumor-mediated metabolic restriction on T cells [18]. Finally, CD26 favors the secretion of Th1 cells and pro-inflammatory cytokines, such as interferon-γ (IFN-γ), interleukin-6 (IL-6), and tumor necrosis factor-α (TNF-α) [17, 19]. Thus, CD26 is a multifunctional molecule involved in many critical immunoregulatory mechanisms, able to modulate several key aspects of lymphocyte function [20, 21]. Furthermore, the strength of CD26 expression on CD4^+^ T lymphocytes permits the characterization of three subtypes with variable degrees of responsiveness to tumors. The CD26^neg^ cells have regulatory properties; the CD26^int^ cells have a naïve-like phenotype, while CD26^high^ cells possess a durable stem memory profile with heightened anti-tumor cytotoxicity [15] with clinical consequences in oncology. In this regard, we posit that CD4^+^CD26^high^ T cells could be a biomarker in cancer management that may be used for the prevention, diagnosis, and selection of therapeutic methods and treatment monitoring of patients in the future.

Given these observations, to our knowledge, the role of CD4^+^CD26^high^ T cells in metastatic melanoma patients has not been detailed. Therefore, our study aimed to investigate for the first time the frequency of circulating CD4^+^ T cells with varying levels of CD26 by flow cytometry in metastatic melanoma patients treated with nivolumab compared to healthy subjects. In addition, the correlation of CD4^+^CD26^+^ T cells with clinical response, disease severity, and survival was also performed.

## Materials and methods

### Patients and human samples

Sixty-nine patients with unresectable stage III or IV melanoma were treated with nivolumab. These patients were prospectively enrolled at the diagnosis of melanoma within the Cancer Immunotherapy and Development Therapeutics Unit of Istituto Nazionale Tumori IRCCS “Fondazione Pascale,” Naples, Italy. The local Ethics Committee approved the study protocol performed according to the Declaration of Helsinki (2000). Each participant provided written informed consent for biological testing. Twenty age- and gender-matched healthy volunteers were recruited as controls. Whole blood samples from melanoma patients were collected before nivolumab therapy (baseline) that was administered via intravenous infusion at the dose of 240 mg or 3 mg/kg every 2 weeks or 480 mg every 4 weeks until disease progression or unacceptable toxicity appeared. Clinical data, such as serum LDH, complete blood count, BRAF status, brain metastasis, and prior lines of treatment, were collected for all patients before nivolumab treatment until the last follow-up. Response assessment was performed at baseline, week 12, and every 12 weeks. The clinical response of the patients was classified according to response evaluation criteria in solid tumors (RECIST) as complete response (CR), partial response (PR), stable disease (SD), or progressive disease (PD) [22]. In addition, twenty age- and gender-matched healthy volunteers were recruited as controls; peripheral blood was collected at the Transfusion Unit of the same Institute.

### Phenotypic analysis of peripheral lymphocyte subsets

Briefly, 50 μL of EDTA-treated whole blood was incubated for 20 min in the dark at room temperature with a cocktail of mAbs, including CD45 (FITC), CD26 (PE), CD3 (PerCP-Cy5.5) CD4 (APC-CY7). Matched isotype mAbs were used as negative controls. All reagents were purchased from Becton Dickinson (BD, San Diego, CA, USA) except for CD26, purchased from BioLegend (USA). After erythrocyte lysis with ammonium chloride (BD FACS Lysing Solution BD Biosciences Pharmingen), samples (5 × 10^4^ events/sample) were acquired on a FACS Canto II Flow Cytometer (Becton Dickinson Immunocytometry Systems, Palo Alto, Calif.). The analysis was performed using the FacsDiva software (Becton Dickinson Immunocytometry Systems, Palo Alto, Calif.). Selection of the lymphocytes containing the gate was based on morphological gating on forward/side light scatters (FSC-A and SSC-A, respectively) and CD45/side scatter. The population of lymphocytes was identified using a gate based on CD3 and CD4 markers. The expression of CD26 was determined on the population of interest CD3^+^CD4^+^ T cells.

### Statistical analysis

Quantitative variables were characterized using mean ± standard deviation (SD) or median with quartile ranges, where appropriate, while categorical factors were described with absolute frequencies and percentages. Accordingly, the comparison between melanoma patients and healthy controls was based on the Mann–Whitney U test. Where appropriate, comparisons were performed with the Wilcoxon test for paired data. Receiver operating characteristic (ROC) analysis was performed to identify the optimal cut-off value to group patients in those with low or high values. Differences in the expression of CD26^high^, according to clinical benefit, Disease Control Rate (DCR) were analyzed by the Mann–Whitney U test while the best overall response rate (BORR) were analyzed by the Kruskal–Wallis rank test (two-sided). Bonferroni adjustment was used for pairwise comparisons. Survival was calculated using Kaplan–Meier curves and compared by the log-rank test. The Cox proportional hazards model calculated the adjusted hazard ratios (HRs) and their 95% confidence interval (CI). The OS time was calculated from the date of diagnosis to the date of death or censored at the last follow-up (FU) visit. The Progression Free Survival (PFS) was calculated from the date of randomization to the first recorded disease progression or the date of death due to any cause.

All tests were two-tailed, and statistical significance was set at p < 0.05. Statistical analyses were made using the GraphPad Prism 7 (GraphPad Software Inc., San Diego, CA, USA) or the MedCalc (MedCalcSoftware, Ostend, Belgium) platforms.

## Results

### Patients’ characteristics

A total of 69 melanoma patients treated with nivolumab were included. The baseline characteristics are listed in Table 1. The median age was 65.5 years; 42 patients were male, and 27 were female. According to the 8th edition of the American Joint Committee on Cancer (AJCC) [2], 12 patients were assigned to category M1a, 15 to M1b, 31 to M1c, and 11 to M1d. BRAF mutational status was known in 56 out of 69 melanoma patients, in particular: 24 patients (42.85%) had BRAF-mutated melanoma while 32 patients (57.14%) were wild-type for BRAF. Brain metastasis was present in 11/69 patients (15.94%). Fifty-four out of 69 subjects received nivolumab as first-line therapy; the remaining patients previously had the administration of ipilimumab or BRAF inhibitor. The best overall response rate (BORR) to nivolumab was 31.88% (22/69): 6 patients with CR and 16 with PR. The DCR to nivolumab was 60.86% (42/69): 6 patients with CR, 16 PR, and 20 with SD). 39.13% of patients (27/69) displayed PD (Table 1).

**Table 1:** Baseline patient and disease characteristics of the study population

	Melanoma patients N = 69		Healthy Controls N = 20	
	N	%	N	%
Age				
Median, years^#^	63		66	
Range	21–87		26–75	
Gender				
Male	42	60,9	13	65
Female	27	39,1	7	35
BRAF mutation				
No	33	47,9	NA	NA
Yes	25	36.2	NA	NA
Not available	11	15,9		
Line of treatment				
1	55	79,7	NA	NA
2 and 3	14	20,3	NA	NA
Distant metastasis				
M1a	12	17,4	NA	NA
M1b	15	21,7	NA	NA
M1c	31	45	NA	NA
M1d	11	15,9	NA	NA
LDH				
Normal	43	62,3	NA	NA
Upper limit of normal	25	36,2	NA	NA
Not available	1	1,5		
Stage				
IIIC	2	2,9	NA	NA
IV	67	97,1	NA	NA

Melanoma patients with CR, PR, or SD greater than 12 months (35/69 patients) were arbitrarily assigned to a group with clinical benefit (CB), while the no-clinical benefit group (NCB) showed PD or SD for lesser than 12 months (34/69 patients). The median follow-up for this study was 14.10 months. Twenty age- and gender-matched healthy volunteers were recruited as controls.

### Frequencies of peripheral CD4^+^CD26^high^ T cells are significantly reduced in melanoma patients

**Fig. 1:**
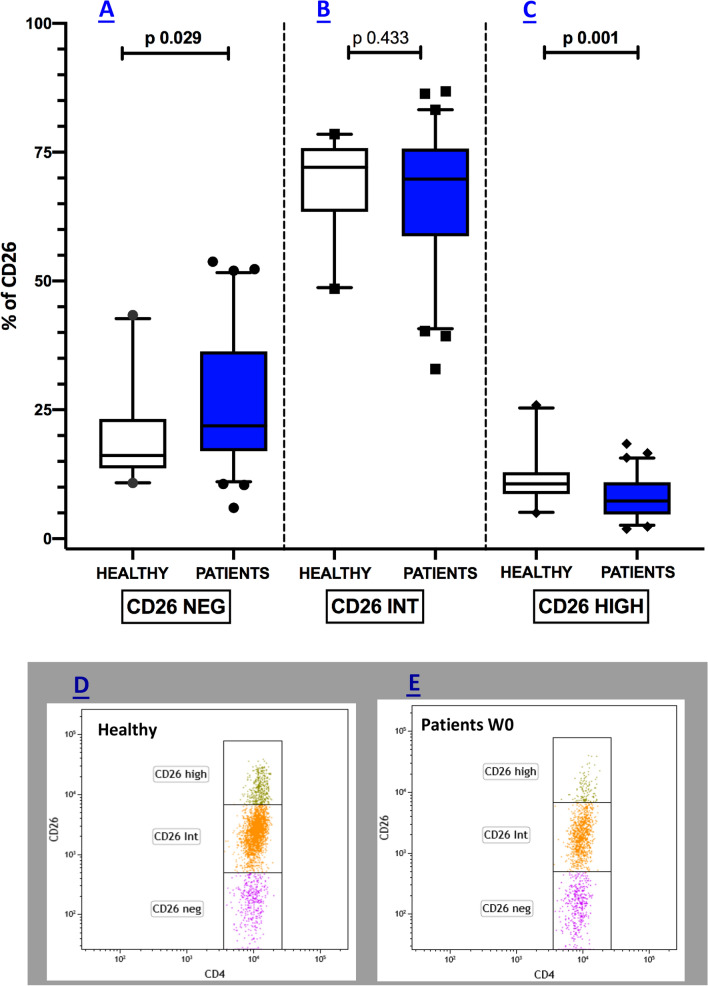
Peripheral CD4^+^CD26^high^ are markedly reduced in melanoma patients. Box plots showing the median (25th; 75th percentile) percentage of CD26^neg^ (**A**), CD26^int^ (**B**), and CD26^high^ (**C**) subsets in healthy subjects and melanoma patients. Representative dot plots illustrating CD26 subsets in healthy and melanoma patients are depicted in **D** and **E** respectively

Flow cytometry revealed the frequency of CD4^+^ T lymphocytes expressing CD26 in healthy versus cancer patients in our study cohort are reported in Fig. 1. The relative median values [25th; 75th percentile] for the different CD4^+^CD26^+^ T cell subsets before starting nivolumab treatment (W0) were respectively 16.15 [13.65; 23.25] for CD4^+^CD26^neg^ (Fig. 1A), 72.05 [63.43; 75.83] for CD4^+^CD26^int^ (Fig. 1B), and 10.65 [8.65; 12.90] for CD4^+^CD26^high^ (Fig. 1C) in healthy individuals. In patients with melanoma, the relative median percentages were 22.40 [16.95; 37.15] for CD4^+^CD26^neg^ (Fig. 1A), 69.50 [58.70; 75.55] for CD4^+^CD26^int^ (Fig. 1B), and 7.30 [4.70; 10.90] for CD4^+^CD26^high^ (Fig. 1C). As reported, the median percentages of CD4^+^ CD26^neg^ T cells, which possess immunosuppressive properties, were elevated in melanoma subjects as compared with healthy individuals (p = 0.029) (Fig. 1A). At the same time, no differences were detected when looking at the naïve-like CD4^+^CD26^int^ percentages (p = 0.433) (Fig. 1B). Of note, the median frequencies of the CD4^+^ T cell expressing CD26^high^ were significantly less in melanoma patients than in the healthy group (p = 0.001) (Fig. 1C).

No differences in the distribution of CD4^+^CD26^high^ were detected through the stratification of melanoma patients according to gender, BRAF mutational status, brain metastasis, and M category (data not shown).

No statistical difference of total lymphocyte counts were found in melanoma patients as compared with healthy controls (p = 0.13).

### CD4^+^CD26^high^ T cells are elevated in patients responsive to nivolumab therapy

**Fig. 2:**
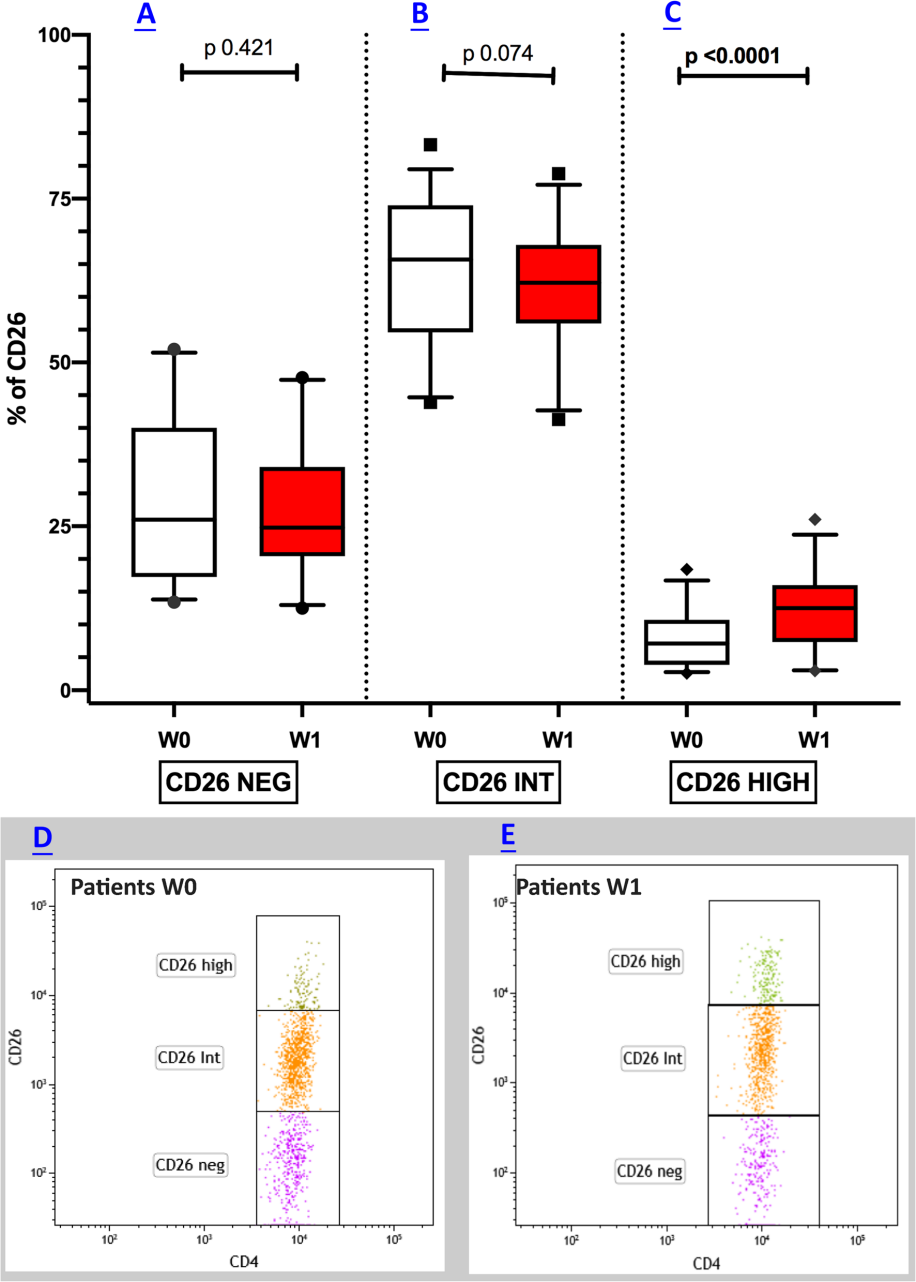
Nivolumab therapy modifies the alterations of CD26^high^ frequencies. Box plots showing the median (25th; 75th percentile) distribution of CD26^neg^ (**A**), CD26^int^ (**B**), and CD26^high^ (**C**) in a sub-cohort of 33 melanoma patients at baseline and after 12 weeks (W1) of nivolumab treatment. Representative dot plots illustrating CD26 subsets melanoma patients at W0 and W1 are depicted in **D** and **E** respectively

To address whether Nivolumab may affect the immune perturbations observed in metastatic melanoma patients, we compared the frequency of CD4^+^CD26^+^ T cells at W0 with those obtained after 12 weeks (W1) of treatment in a sub-cohort of 33 patients (Fig. 2A–C). The analysis showed that at W1 median (25th; 75th percentile) percentages of CD4^+^CD26^high^ T cell subsets were significantly higher in comparison with those measured before starting Nivolumab treatment. In particular, we found that CD4^+^CD26^high^ T cells rose from 7.1 (3.89; 10.70) to 12.5 (7.34; 16.00) (p < 0.0001), as displayed in Fig. 2C.

The manifold increases from basal to W1 values were 5.4-fold (+ 76.00%) for CD4^+^CD26^high^ T cells, nearly aligning the cell subset values with the ranges seen in the blood of healthy controls.

No statistical differences of total lymphocyte counts were found between W0 and W1 melanoma patients (p = 0.45).

### Fewer CD4^+^CD26^high^ T cells in the blood are associated with reduced patient survival

**Fig. 3:**
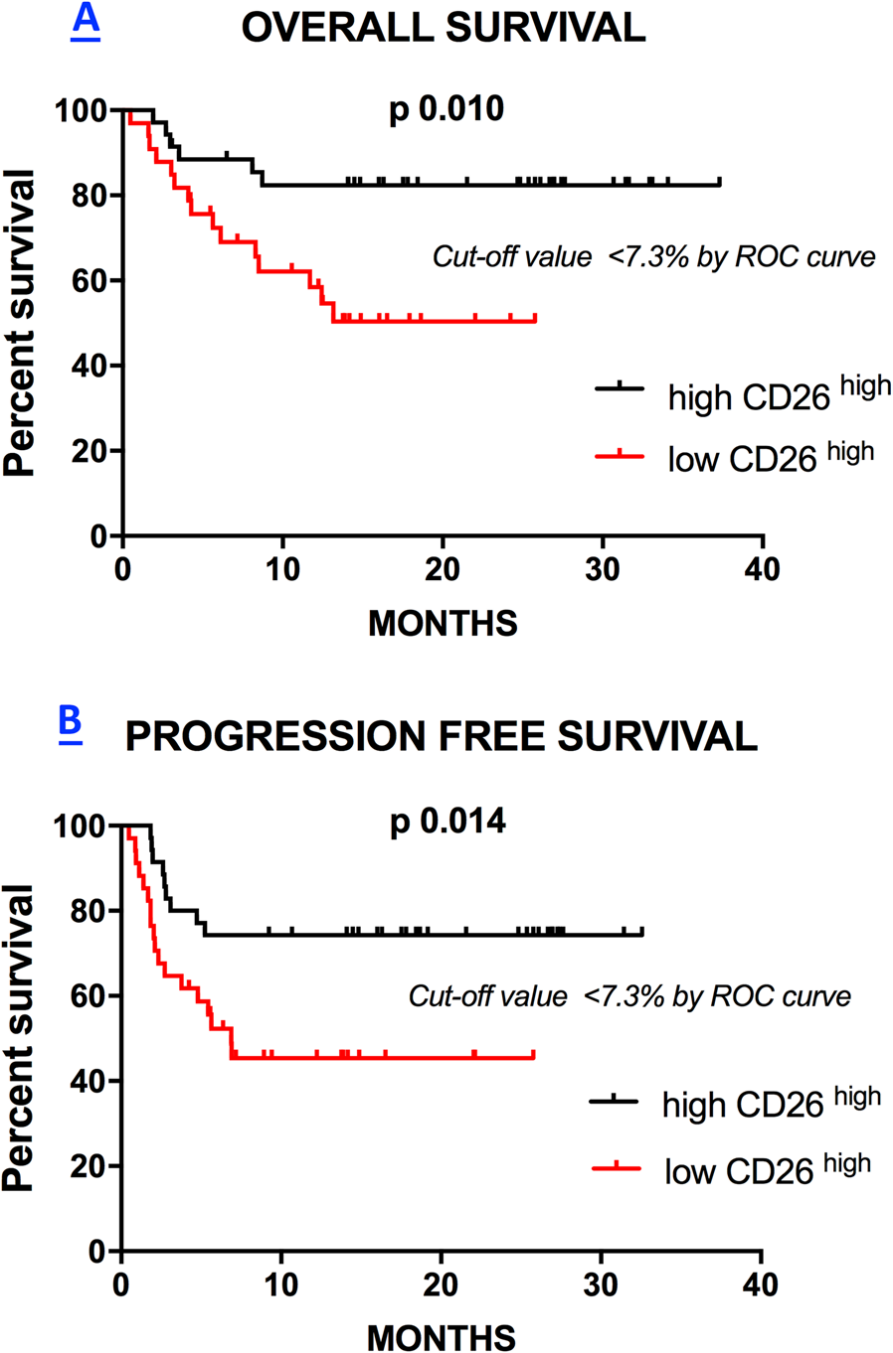
Low frequencies of CD26^high^ levels are associated with reduced survival in melanoma patients. Kaplan Meier curves representative of the Overall Survival (OS) (**A**) and Progression Free Survival (PFS) (**B**) of melanoma patients according to the high (> cut-off) or low (< cut-off) baseline levels (W0) of circulating CD26^high^

A log-rank test was performed for 69 metastatic melanoma patients (the whole cohort of melanoma patients) to investigate the association between the frequencies of peripheral CD4^+^CD26^+^ T cells and the OS. Results showed that melanoma patients with lower frequencies of CD4^+^CD26^high^ T cells (cut-off value of < 7.3%, identified by ROC curve) had significantly worse survival outcomes (p 0.010) (median survival > 25.77 months; HR 3.31; 95% CI 1.35 to 7.62) as compared with patients with higher CD4^+^CD26^high^ percentages (median survival > 37.3 months; HR 0.32; 95% CI 0.13 to 0.76) (Fig. 3A). Conversely, no significant associations with patient outcomes were found with CD4^+^CD26^neg^ and CD4^+^CD26^int^ frequencies (data not shown).

Lastly, we investigated the association between CD4^+^CD26^+^ T cell frequency in the blood of patients and PFS. Interestingly, we found that a low percentage of CD4^+^CD26^high^ lymphocytes in the blood (cut-off value of < 7.3%, identified by ROC curve) were significantly associated with worse prognosis (p = 0.014) (median survival 6.87 months; HR 2.59; 95% CI 1.20 to 5.56) when compared to patients with more CD4^+^CD26^high^ T cells in the blood (median survival > 32.57 months; HR 0.38; 95% CI 0.17 to 0.82) (Fig. 3B). Again, no significant associations were found with CD4^+^CD26^neg^ and CD4^+^CD26^int^ frequencies and PFS (data not shown).

### Correlation of baseline CD4^+^CD26^high^ T cells frequencies and clinical response to nivolumab

**Fig. 4:**
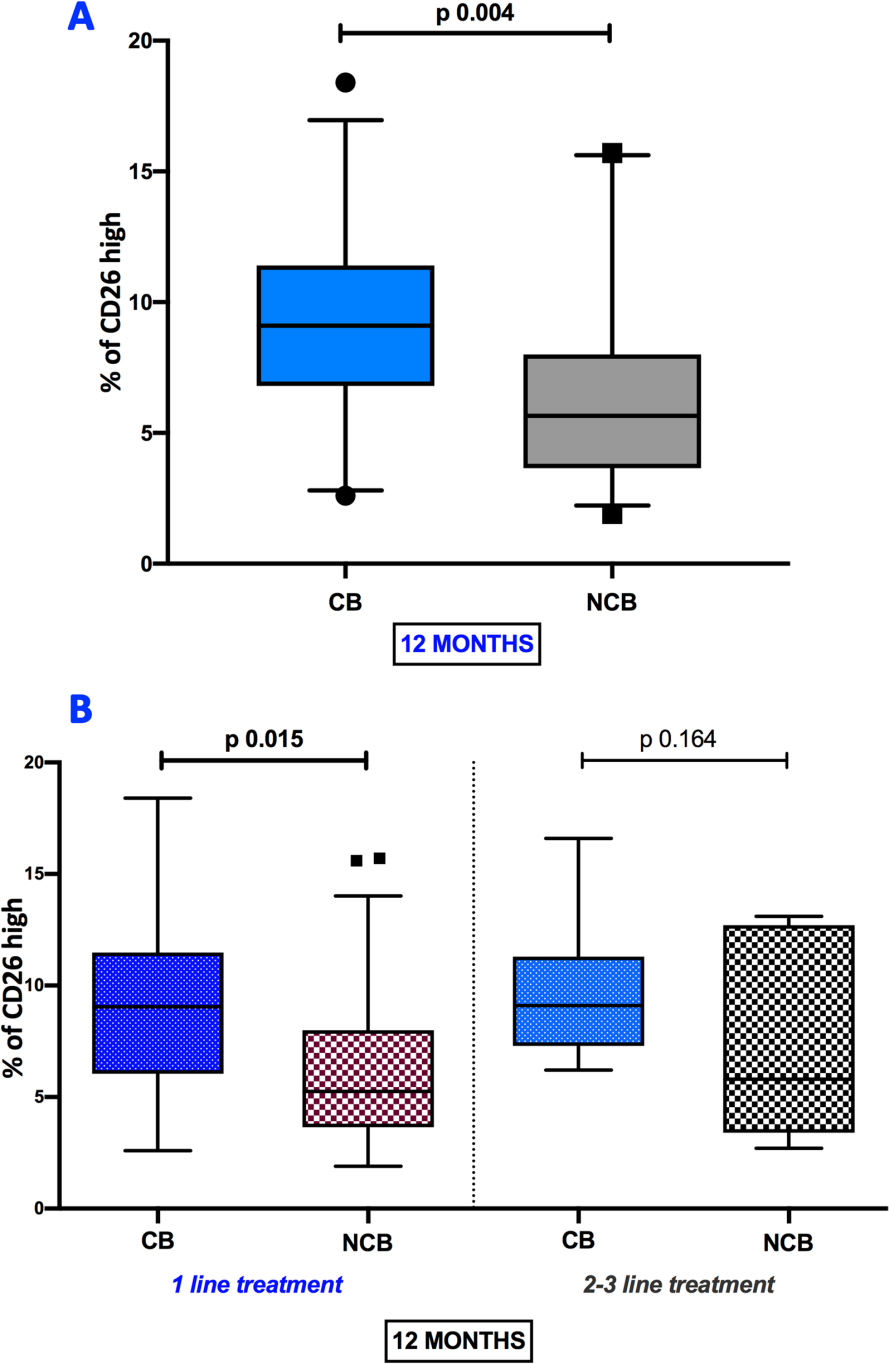
Correlation between percentages of CD4^+^CD26^high^ and clinical benefit to nivolumab. Box plots showing the median (25th; 75th percentile) levels of CD26^high^ cells in melanoma patients stratified in clinical benefit (CB) e no CB (NCB) groups at 12 months (**A**). Box plots showing the median (25th; 75th percentile) baseline frequencies of CD26^high^ lymphocytes in CB and NCB subgroups of patients treated with 1st line nivolumab and 2nd/3rd line nivolumab at 12 months (**B**)

Melanoma patients were arbitrarily stratified into two groups according to clinical response to nivolumab therapy, as indicated in the Material and Methods paragraphs, to assess the correlation between CD4^+^CD26^high^ T lymphocytes and clinical benefit to nivolumab. In this regard, Mann–Whitney test showed that median frequencies of the CD4^+^ T cell expressing high CD26 were significantly higher in the CB group as compared with the NCB group (9.1 [6.8; 114] versus 5.65 [3.6; 8.0] respectively) (p 0.004) at 12 months (Fig. 4A). According to these data, the association between CD4^+^CD26^high^ T lymphocytes and clinical benefit at 12 months in patients treated with 1st line nivolumab and 2nd/3rd line nivolumab was also assessed. The median frequencies of CD4^+^CD26^high^ T lymphocytes in patients with CB under 1st line nivolumab treatment were higher than frequencies observed in NCB patients with 1st line nivolumab (9.05 [6.05; 11.48] versus 5.25 [3.65; 8.0] respectively) (p 0.015) as shown in Fig. 4B. Among the patients under treatment with 2nd/3rd line nivolumab, no significant difference of CD4^+^CD26^high^ T cells frequency was observed between CB and NCB patients (Fig. 4B). These data show that high pre-treatment CD4^+^CD26^high^ T levels are significantly correlated with clinical response in patients given nivolumab treatment.

**Fig. 5:**
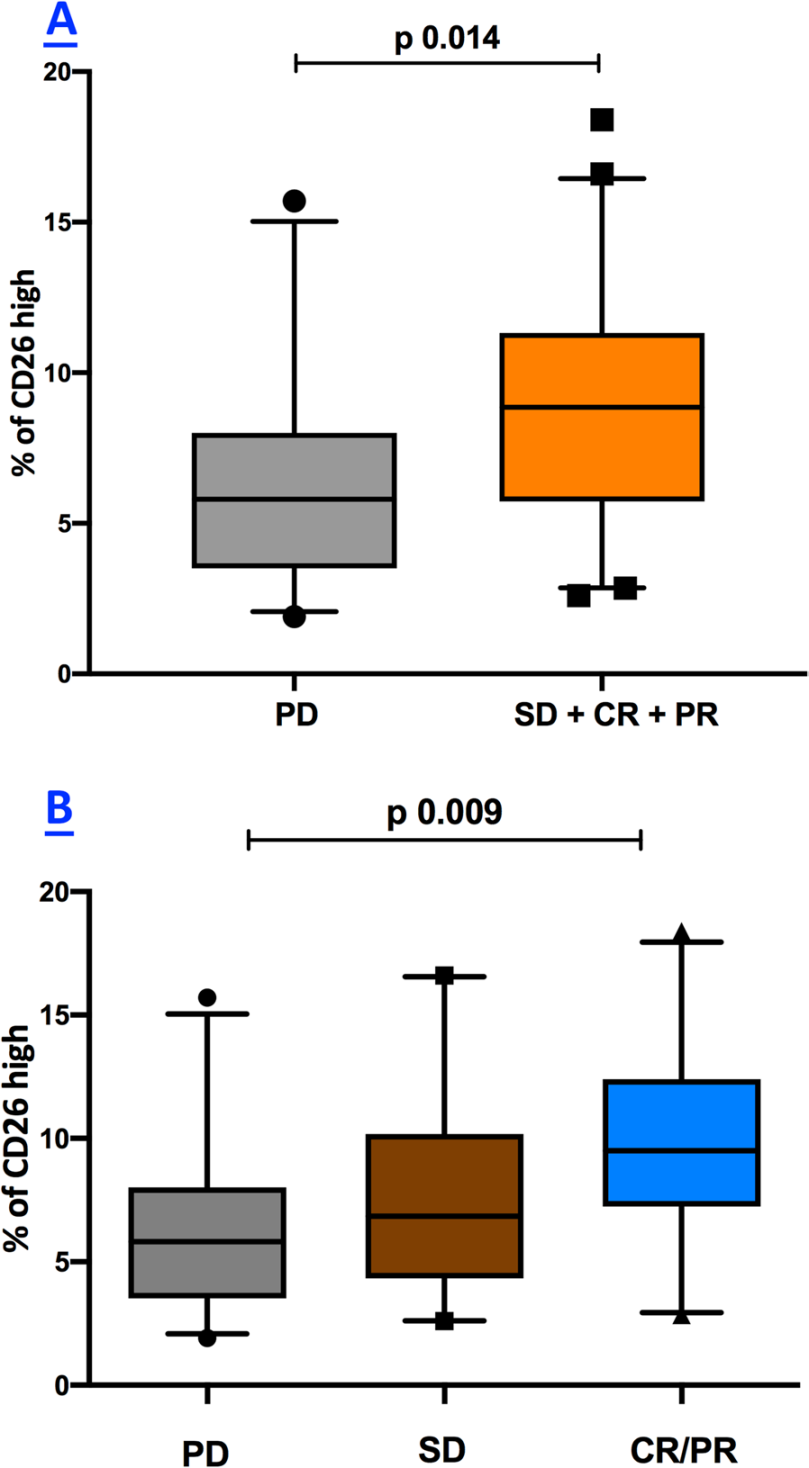
Relationship between baseline levels CD4^+^CD26^high^ and treatment response to nivolumab. Box plots showing the correlation between median (25th; 75th percentile) levels of CD26^high^ cells, DCR (**A**), and BORR (**B**) of melanoma patients under treatment of nivolumab at 12 months

Successively we evaluated the predictive value of baseline CD4^+^CD26^high^ T cells to the treatment response. In this context, the DCR of melanoma patients under treatment of nivolumab at 12 months (patients with CR, PR, and SD vs. PD) was significantly correlated with a higher pre-treatment median proportion of circulating CD4^+^CD26^high^ T cells (8.85 [5.72; 11.33]) in patients with CR, PR, and SD than median occurring in patients with PD (5.80 [3.51; 8.00]) p 0.014, (Fig. 5A). In line with these data also, the BORR of nivolumab-treated patients at 12 months (CR/PR vs. SD vs. PD) was significantly correlated with a higher pre-treatment median proportion of circulating CD4^+^CD26^high^ T cells (9.50 [7.24; 12.40]) in patients with CR/PR than median occurring in patients with SD (6.85 [4.32; 10.18]) and PD (5.80 [3.51; 8.00]) p 0.009 (Fig. 5B).

## Discussion

To the best of our knowledge, this is the first clinical report addressing the frequency of CD4 + T cell subsets expressing CD26 in the setting of metastatic melanoma patients under nivolumab immunotherapy.

Our results show that circulating CD4^+^CD26^high^ T is reduced in metastatic melanoma patients at baseline. However, However, the few CD4^+^CD26^high^ T lymphocytes in patients are induced after 12 weeks of nivolumab treatment, with manifold increases to a similar range as that detected in healthy controls. Data obtained also demonstrate a significant association between low percentages of blood circulating CD4^+^CD26^high^ T cells and the reduced survival of metastatic melanoma patients with stage III-IV. Interestingly, the baseline frequencies of peripheral CD4^+^CD26^high^ lymphocytes in melanoma patients under ICIs therapy were associated with clinical benefit. Mainly, patients with high baseline proportions of circulating CD4^+^CD26^high^ T cells had good clinical outcomes from nivolumab treatment. Therefore, our results reveal a role of blood circulating CD4^+^CD26^high^ T cells as potential biomarkers to predict the anti-PD-1 response in melanoma patients.

Cumulative evidence has shown that CD26 plays a crucial role in immune regulation as a molecule capable of transmitting T-cell activation signals and regulating chemokine [16, 17]. Accordingly, one possible result of inflammatory processes appears to be an upregulation of the CD26 expression as an activation marker for T cells [23, 24]. Moreover, increased CD26^+^CD4^+^ T cells have been observed in subjects affected by immune-mediated disorders, including autoimmune diseases and graft-versus-host disease (GVHD) [25–27]. In this regard, several reports have described a CD4^+^ memory T cells subset expressing CD26 molecule in the peripheral blood of healthy donors. The CD4^+^CD26^+^ T cell subset can respond maximally to recall antigens [16, 17, 28] and is functionally correlated with T cell signal activation [16, 17, 29]. Consequently, this body of previous literature supports our observation that high CD26 expression on T cells is a marker of patient responsiveness to ICI therapy.

In line with these previous data, our team recently reported that three subtypes of human CD4^+^ T cells with varying responsiveness to tumors could be distinguished through CD26 expression. In particular, the CD26^neg^ had regulatory properties, the CD26^int^ had naïve/central memory phenotype, and CD26^high^ was cytotoxic and resistant to apoptosis with an increased chemokines receptor expression and durable stem memory profile [30]. As a clinical consequence, CD26^high^ T cells engineered with a CAR could eradicate large human tumors with greater effectiveness of Th17, Th1, or Th2 cells. In particular, CD4^+^CD26^high^ T lymphocytes could secrete effector cytokines, produce cytotoxic molecules, and persist long-term [15, 30]. These properties license CD4^+^CD26^high^ T cells with a natural capacity to traffic, regress, and survive in solid tumors suggesting that CD26^high^ T lymphocytes played a fundamental role in host immune response. In particular, mice bearing melanoma, mesothelioma, or pancreatic cancer experienced a better treatment outcome when a high percentage of CD26^+^ donor T cells infiltrated the tumor, suggesting that a CD26 played a crucial role in tumor immunity, which could entail several mechanisms [30]. Interestingly, a significant reduction in the serum CD26 activity and the percentage of CD26^+^ lymphocytes were found in patients with melanoma compared to healthy subjects [31].

Herein, we clearly show, for the first time, that the frequencies of blood circulating CD26^high^ CD4 + T lymphocytes are significantly reduced in metastatic melanoma patients. Our data are noteworthy if we consider that enumeration and phenotypic analysis of CD26^high^ suffer from their low percentages at the peripheral level, even in healthy individuals. Our hypothesis to be investigated is that the natural diminishment of CD4^+^CD26^high^ T lymphocytes that we observed in our melanoma cohort may be likely attributable to the immune suppression factors induced by metastatic melanoma cancer or alterations in tissue recruitment of lymphocytes homing induced by changes in chemokine gradients. In this regard, malignant cells can secrete immunosuppressive factors such as Tumor growth factor (TGF)-beta1 derived soluble factors that down-regulate CD26/DPP4 expression in T cells leading to reduced serum CD26/DPP4 activity in cancer patients [32]. Also, CD26^high^ T cells can carry out a trans-endothelial migration, inducing both enzymatic cleavages of chemokines that regulate migration and co-stimulation via caveolin-1 that could affect T cell function [17].

Several recent studies have reported that a higher frequency of circulating central memory T cells (CD4 and CD8) is associated with an increased tumor inflammatory profile in melanoma patients and with longer survival times [33–35]. According to these data, more memory T cells in baseline Peripheral Blood Mononuclear Cells (PBMC) are a possible biomarker candidate to predict clinical response to anti-CTLA-4 treatment in advanced melanoma patients [36]

In line with these previous data, the high frequencies of blood circulating CD26^high^ T lymphocytes in our present study strongly correlate with clinical responses. In addition, higher baseline levels of the CD4^+^CD26^high^ T cells of the peripheral blood are significantly associated with the clinical benefit. Notably, this result was much more evident in patients undergoing the first-line treatment of nivolumab, as compared to patients treated with second/third line of nivolumab, suggesting that the Nivolumab treatment may modulate the expression of CD26 on T cells.

Furthermore, the baseline proportion of these cells in the peripheral blood was strongly and consistently correlated with patients' DCR and BORR during nivolumab treatment.

Altogether, these results are intriguing as the baseline levels of CD4 + T cells, expressing high levels of CD26, may be a predictive marker for treatment response to nivolumab in metastatic melanoma patients. Interestingly, in our study, additional proof that further reinforces the role of CD26^high^ as a biomarker in metastatic melanoma patients is the finding that lower baseline levels of CD4^+^CD26^high^ T cells were correlated significantly with reduced OS and PFS in melanoma patients.

Lastly, to address whether the immune-inflammatory perturbations we observed in melanoma patients at baseline are influenced by currently available nivolumab treatment, we measured the frequency distribution of circulating CD4^+^CD26^high^ levels in a sub-cohort of 33 cases. We found that ICI treatment can exert a modulating effect at 12 weeks from the initiation of treatment. Levels of the CD4^+^CD26^high^ T cells were increased during nivolumab treatment, aligning the cell subset values with the ranges of healthy controls.

Based on the results of our study, frequencies of circulating CD4^+^CD26^high^ cells may have predictive implications for outcome after treatment with nivolumab and prognostic implications for metastatic melanoma patients in general, regardless of therapy.

As literature data are lacking in this setting, we suppose our results are only a benchmark because of the relatively small cohort of patients analyzed for ICI therapy. From this point of view, the small sample size and the lack of a validation cohort represent a limitation of our study that necessarily requires the confirmation of our results in larger patient cohorts. Therefore, in future studies, we plan to further validate the baseline level of CD26^high^ T lymphocytes as a biomarker in a larger, prospectively followed cohort.

## Conclusion

This report firstly demonstrates a significant reduction of CD4^+^CD26^high^ T lymphocytes in metastatic melanoma patients. Our study identifies CD26^high^ T cells as potential biomarkers whose perturbations are associated with reduced survival and with worse clinical outcomes of the patients. Deciphering the involvement of the immune system in the pathogenesis of melanoma has become a prosperous field of exploration for therapeutic purposes in recent years. However, even though the immune system plays a crucial role in melanoma biology, there is little use of immune biomarkers to evaluate the host’s response to cancer that could identify several mechanisms to track successful or failed therapy [37].

Our results are promising to reveal new scenarios both in terms of a better understanding the biological mechanisms underlying the disease and identifying easy-to-perform biomarkers predictive of clinical behavior in patients.

## Data Availability

Data are available in a public, open-access repository https://zenodo.org/record/7588930#.Y9js_nbMKUk.

## Abbreviations

ICIs: Immune checkpoint inhibitors
CTLA-4: Cytotoxic T-Lymphocyte Antigen 4
PD-1: Programmed death-1
PD-L1: Programmed death-ligand 1
OS: Overall survival
mAb: Monoclonal antibody
TIL: Tumor-infiltrating lymphocytes
CAR: Chimeric antigen receptors
DPP4: Dipeptidyl peptidase 4
ADA: Adenosine deaminase
Th: T helper
IFN-γ: Interferon-γ
IL-6: Interleukin-6
TNF-α: Tumor necrosis factor-α
LDH: Lactate dehydrogenase
RECIST: Response evaluation criteria in solid tumors
CR: Complete response
PR: Partial response
SD: Stable disease
PD: Progressive disease
SD: Standard deviation
ROC: Receiver operating characteristic
HRs: Hazard ratios
PFS: Progression free survival
CI: Confidence interval
FU: Follow-up
AJCC: American Joint Committee on Cancer
BORR: Best overall response rate
DCR: Disease control rate
CB: Clinical benefit
NCB: No-clinical benefit
GVHD: Graft-versus-host disease
PBMC: Peripheral blood mononuclear cell
